# Dietary Chinese herbal preparations improve growth performance, immune function, and gut health in weaned piglets

**DOI:** 10.3389/fvets.2026.1911773

**Published:** 2026-09-07

**Authors:** Shan Yang, Zijie Zhang, Hongzhan Cao, Chunlian Lu

**Affiliations:** College of Animal Science and Technology, Agricultural University of Hebei, Baoding, Hebei, China

**Keywords:** Chinese herbal medicine additive, growth performance, immune function, piglets, antioxidant

## Abstract

**Introduction:**

The search for effective antibiotic-free feed additives is critical for sustainable swine production. Compound Chinese herbal additives (CCHA) have attracted interest due to their potential bioactivity, but their optimal feeding concentration and duration for weaned piglets remain unclear.

**Methods:**

A total of 210 healthy piglets aged 28 ± 1 days were randomly assigned to seven groups (control group-CON; 0.2%‑14d; 0.3%‑14d; 0.4%‑14d; 0.2%‑28d; 0.3%‑28d; 0.4%‑28d), with three replicates per group. Data from the six treatment groups were analyzed using two‑factor ANOVA within a general linear model, while one‑way ANOVA was applied for comparisons across all seven groups. Growth performance, nutrient digestibility, serum biochemical and immunological parameters, antioxidant capacity, and fecal microbiota composition were measured.

**Results:**

Dietary CCHA supplementation significantly increased average daily gain (ADG) and average daily feed intake (ADFI) compared with CON (p < 0.01), though ADFI declined with extended feeding (*p* < 0.01). Crude protein (CP) digestibility improved (*p* < 0.05), while dry matter (DM) digestibility was significantly affected by feeding duration (*p* < 0.01). Serum total protein (TP), albumin (ALB), blood urea nitrogen (BUN), immunoglobulins, and total antioxidant capacity were all elevated in treated groups (*p* < 0.05). Prolonged feeding (28 d) further enhanced ADFI, CP and crude ash (ASH) digestibility, serum protein, immunoglobulin G (IgG), and total antioxidant capacity (T‑AOC), while reducing alanine transaminase (ALT) activity (*p* < 0.05). However, optimal concentrations varied: 0.2% favored growth, 0.3% benefited digestion and protein metabolism, whereas 0.4% showed inhibitory effects under long‑term feeding. Microbiota sequencing revealed increased abundances of Lactobacillus and Clostridiaceae in CCHA‑supplemented piglets.

**Conclusion:**

These findings indicate that CCHA can effectively improve growth performance, nutrient utilization, immune status, antioxidant capacity, and gut microbial composition in weaned piglets, thereby positioning it as a promising antibiotic‑free feed additive.

## 1 Introduction

Since the advent of the “antibiotic-free” era, an increasing number of researchers have been dedicated to identifying and developing alternatives to antibiotics or environmentally friendly, non-toxic feed additives in order to eliminate or mitigate the negative impacts of banned antibiotics. Chinese herbal preparations have made a significant contribution to animal health. As all-natural products, they are characterized by minimal side effects, the absence of residues, and a low risk of developing drug resistance, and have attracted considerable attention from the livestock industry in recent years. The active components of Chinese herbal medicines are complex and diverse, primarily including polysaccharides, flavonoids, saponins, terpenoids, essential oils, alkaloids, and organic acids. These active compounds help improve the production performance and immune function of piglets (1).

Herbal feed additives contain a high concentration of organic compounds and trace elements, which can enhance the growth performance of weaned piglets, improve carcass quality, boost the reproductive performance of breeding pigs, and enhance fattening efficiency (2, 3). Feeding weaned piglets a compound herbal preparation containing *Agastache*, *Perilla*, and *Angelica dahurica* has a significant effect on increasing average daily gain (ADG) and reducing the feed-to-gain ratio (F/G) (4). The administration of traditional Chinese herbal preparations to sows in Ningxiang not only improves their reproductive performance and lactation capacity but also enhances the growth performance of piglets (5). Research by Wang (6) indicates that adding a 1% concentration of a compound herbal formula consisting of 10 ingredients—including *Codonopsis*, *Astragalus*, *Honeysuckle*, and *Angelica*—to the diet of finishing pigs can improve their growth performance, slaughter performance, and meat quality. Jiang and colleagues (7) demonstrated that adding a compound Chinese herbal additive, comprising primarily astragalus, goji berries, and hawthorn, to the finishing pigs’ diet can effectively improve growth rates and fattening performance, while also enhancing the pigs’ immune function and antioxidant capacity.

An animal’s resistance to disease depends on the strength of its immune system, which is primarily reflected in the levels of serum antibodies, cytokines, and complement proteins. There are numerous reports on the immune-boosting effects of Chinese herbal medicines. Adding compound Chinese herbal preparations at various concentrations to broiler chicken diets has been shown to significantly increase serum immunoglobulin and complement levels, while also significantly elevating the mRNA expression levels of the cytokines IL-2 and IFN-*γ* (8). Yupingfeng extract promotes the development of immune organs in broiler chickens. It exerts its effects by regulating the function of the NF-κB immune and inflammatory signaling pathway in the spleen and liver, thereby promoting the synthesis of anti-inflammatory factors and inhibiting the production of downstream inflammatory factors, which in turn reduces inflammation and enhances the chickens’ immunity (9). Berberine can inhibit the NF-κB/LCN2 pathway in the hippocampus of mice, reduce the production of inflammatory cytokines, and thereby alleviate the inflammatory response (10). During biological processes, the body’s metabolism produces free radicals. Under normal conditions, the production and elimination of free radicals are in dynamic equilibrium; however, if the internal or external environment of the body is subjected to stress or undergoes changes, this dynamic equilibrium is disrupted (11). The excessive production of free radicals within the body can lead to oxidative damage and tissue disorders, which in turn affects animal productivity (12). The flavonoids found in traditional Chinese medicines such as *Scutellaria baicalensis* and *Forsythia suspensa* possess potent antioxidant activity, capable of scavenging reactive oxygen species (ROS) in the body and activating host antioxidant enzymes (13). Berberine, an alkaloid found in *Coptis*, has been shown to increase superoxide dismutase (SOD) activity and reduce malondialdehyde (MDA) levels (14).

This study used *Coptidis Rhizoma*, *Scutellariae Radix*, *Lonicerae Japonicae Flos*, *Ephedrae Herba*, *Armeniacae Semen Amarum*, *Atractylodis Macrocephalae Rhizoma*, *Codonopsis Radix*, *Forsythiae Fructus*, *Poria*, etc., as well as probiotics as the main ingredients of compound Chinese herbal additives (CCHA). This formula fully leverages the complementary benefits of several herbs; when used in combination, they are more effective than when used individually. It has the effects of clearing heat and detoxifying, cooling the blood and resolving stasis, tonifying the spleen and kidneys, and promoting diuresis to reduce swelling (15). The purpose of this study is to investigate the effects of this compound formulation on the growth performance, blood biochemistry and hematology parameters, immune and antioxidant capacity, and gut health of weaned piglets.

## 2 Materials and methods

### 2.1 Animal care

The study was conducted in accordance with the Committee on Laboratory Animal Management and Ethics at Hebei Agricultural University, Hebei Province (protocol code 2026219 and approval date: March 17,2026).

### 2.2 Animals, diets, and experimental design

The main components of CCHA are fermented products of 18 traditional Chinese medicines including *Coptidis Rhizoma*, *Scutellariae Radix*, *Lonicerae Japonicae Flos*, *Ephedrae Herba*, *Armeniacae Semen Amarum*, *Atractylodis Macrocephalae Rhizoma*, *Codonopsis Radix*, *Forsythiae Fructus*, *Poria*, etc., as well as probiotics. The formula was purchased from Shijiazhuang Baicao Mule Biotechnology Co., Ltd., as a commercialized finished product of fermented traditional Chinese medicine named Yuanlankang.

The diet formulation and nutritional composition are shown in Table 1.

**Table 1:** Composition and nutrient levels of basal diets(air-dry basis)

Itemts	Content(%)
Ingredients	
Corn	63.20
Soybean meal(CP37%)	19.00
Whey powder	4.80
Fish meal	8.60
Glucose	1.00
Acidifier	0.30
CaHPO4	0.60
Limestone	0.70
NaCl	0.30
L-Lys•HCL	0.30
DL-Met	0.10
L-Thr	0.10
Premix a	1.00
Total	100.00
Chemical composition	
DE/(Mcal/kg)b	3.25
CP	18.60
SID Lys	1.12
SID M+C	0.69
SID Thr	0.79
SID Trp	0.19
Ca	0.82
Total P	0.65

a. The acidifier used was citric acid.

b. Premix provided the following per kg of the diet: 12,500 U Vitamin A; 1250 U Vitamin D; 125 U Vitamin E; 90 μg Vitamin B12; 10 mg riboflavin; 48 mg pantothenic acid; 35 mg niacin; 4.5 mg folic acid; 0.25 mg biotin; 130 mg Fe; 180 mg Zn; 15 mg Cu; 30 mg Mn; 0.60 mg I; 0.25 mg Se.

c. The chemical composition was a calculated value.

In this experiment, 210 healthy pigs aged 28 ± 1 days were selected and randomly divided into 7 groups after weaning (with an equal male-to-female ratio). The groups were a control group CON (diet containing no CCHA or antibiotics), and six experimental groups. Group I, II, and III received diets containing 0.2, 0.3, and 0.4% of the preparation for 14 days, respectively; and Group IV, V, and VI were given 0.2, 0.3, 0.4% levels, respectively, for 28 days (Table 2). Each group consisted of 3 replicates, with 10 pigs per replicate. The total experimental period lasted 31 days, comprising a 3-day adaptation phase followed by a 28-day formal trial. Groups I, II, and III received the experimental diet for the first 14 days after adaptation and then were switched to the basal diet for the remaining 14 days; in contrast, groups IV, V, and VI were fed the experimental diet continuously throughout the entire 28-day trial period. During the 3-day adaptation period, all piglets were fed the basal diet ad libitum. Samples were collected 3 days before the end of the experiment. Before the commencement of the experiment, the pig house was thoroughly disinfected. Throughout the trial, piglets were provided with ad libitum access to feed and water and were vaccinated according to standard routine immunization protocols. Additionally, the pens were regularly disinfected at scheduled intervals during the experimental period.

**Table 2:** Experimental group assignment.

Groups	Adaptation period (1–3d)	Early phase diet (4–17d)	Last phase diet (18–31d)
CON	Basal diet	Basal diet	Basal diet
I		Test diet (0.2%)	Basal diet
II		Test diet (0.3%)	Basal diet
III		Test diet (0.4%)	Basal diet
IV		Test diet (0.2%)	Test diet (0.2%)
V		Test diet (0.3%)	Test diet (0.3%)
VI		Test diet (0.4%)	Test diet (0.4%)

### 2.3 Data and sample collection

The digestion trial was conducted using the total collection method. During the final 3 days of the experimental period, all feces excreted by pigs in each replicate pen were collected at a fixed time daily. The total fresh weight per 24 h was weighed and recorded. After thorough mixing, the feces were subsampled by the quartering method. A portion was dried at 60–65 °C and ground for the determination of apparent digestibility, while another portion was thoroughly mixed with 10% hydrochloric acid, dried at 60–65 °C, and ground for the determination of protein digestibility. One day before the end of the experiment, collect fresh fecal samples from each replicate in every group that have not come into contact with the ground. The inner part of the middle section of each sample was taken using a sterile toothpick, then divided the collected fecal samples into 2 mL cryogenic tubes, with each vial containing 0.5–2 g of feces. Each sample was divided into 2–3 tubes for backup. After packaging, the samples were immediately placed in liquid nitrogen for rapid freezing, to be used for the detection of fecal microbiota indicators. At the end of the experiment, 20 healthy piglets were randomly selected (one per replicate), fasted for 12 h with free acess to water, 10 mL of blood was collected from the anterior vena cava using tubes without anticogulant. The samples were left to stand at room temperature for 15 min, then centrifuged at 3000 r/min to separate the serum, which was stored at −20 °C and analyzed promptly for serum biochemical and immune parameters.

### 2.4 Chemical analysis

#### 2.4.1 Measurement of growth performance

At the beginning and end of the experiment, all piglets were weighed individually after an overnight fast to determine initial body mass (IBM) and final body mass (FBM). Throughout the trial, the amount of feed input and residual feed were recorded daily on a pen basis to calculate actual feed intake.

The following growth performance parameters were calculated based on these measurements:

$\begin{array}{l} \text{Average Daily Gain}\;(\mathrm{ADG})=\\ (\text{Final Body Mass}–\text{Initial Body Mass})/\\ \text{Number of Days in the Trial}. \end{array}$

$\begin{array}{l} \text{Average Daily Feed Intake}\;(\text{ADFI})=\\ (\text{Feed Input Amount}–\text{Residual Feed Amount})/\\ \text{Number of Test Days}. \end{array}$

$\begin{array}{l} \text{Feed}-\text{to}-\text{gain ratio}\;(F/G)=\text{Total feed intake}/\\ \text{Total weight gain}. \end{array}$

$\begin{array}{l} \text{Gain}-\text{to feed ratio}\;(G/F)=\text{Total weight gain}/\\ \text{Total feed intake}. \end{array}$

#### 2.4.2 Apparent digestibility

Moisture content was determined by the 105 °C constant-weight method (AOAC 934.01), and ash content was determined by the loss-on-ignition method (AOAC 942.05). Crude protein was analyzed using the Kjeldahl method (AOAC 991.20), and crude fat was measured by the Soxhlet extraction method (AOAC 920.39). Apparent nutrient digestibility was calculated using the total collection method. Total nutrient intake was calculated based on the ADFI and the nutrient content in the diet, while total nutrient output in feces was calculated based on the daily fecal output and the nutrient content in feces. The calculation formula was as follows:

$\begin{array}{l} \text{Apparent digestibility}\;(\%)=\\ (\text{total nutrient intake}–\text{total nutrient output in feces})/\\ \text{total nutrient intake}\times 100\%. \end{array}$

where total nutrient intake (g/d) = ADFI (g/d) × nutrient content in diet (%); total nutrient output in feces (g/d) = average daily fecal output (g/d) × nutrient content in feces (%).

#### 2.4.3 Serum protein content

The determination of total protein (TP) (A045-2-1), albumin (ALB) (A028-2-1), serum urea (UREA) (C013-2-1), aspartate transaminase (AST) (C010-1-1), alanine transaminase (ALT) (C009-1-1), alkaline phosphatase (ALP) (A059-1-1), acid phosphatase (ACP) (A060-1-1), and creatine kinase (CK) (A032-2-1) were all performed using kits purchased from Nanjing Jiancheng Co., Ltd.

#### 2.4.4 Serum immunological markers

Serological immune markers include immunoglobulin G (IgG) (E026-1-1), immunoglobulin M (IgM) (E025-1-1), immunoglobulin A (IgA) (E027-1-1), interleukin-2 (IL-2) (H003-1-1), interleukin-4 (IL-4) (H005-1-2), interferon-*γ* (IFN-γ) (H025-1-1), and tumor necrosis factor-*α* (TNF-α) (H052-1-1). The assay kits used in the experiment were purchased from Nanjing Jiancheng Co., Ltd.

#### 2.4.5 Serum antioxidant markers

The following serum antioxidant parameters were measured using kits from Nanjing Jiancheng Co., Ltd.: total antioxidant capacity (T-AOC) (A015-5-1), superoxide dismutase (SOD) (A001-1-1), catalase (CAT) (A007-5-2), glutathione peroxidase (GSH-Px) (A005-2-1), and malondialdehyde (MDA) (A003-1-1).

#### 2.4.6 Fecal microbiome

Total microbial DNA was extracted from the samples using the MagPure Soil DNA LQ Kit (D6356-03) from Mo Bio/QIAGEN. DNA concentration was determined by measuring the DNA absorbance at 260 nm and 280 nm using a fluorescence spectrophotometer (Quantifluor-ST fluorometer, Promega, E6090; Quant-iT PicoGreen dsDNA Assay Kit, Invitrogen, P7589), and DNA quality was assessed via 1% agarose gel electrophoresis. The DNA solution concentration was adjusted, with working solution at 4 °C and the stock solution at −20 °C.

For library preparation, the Illumina TruSeq DNA library preparation protocol was followed. Briefly, genomic DNA was fragmented using high-pressure nitrogen gas or a Covaris instrument, and the resulting fragments were repaired to blunt ends using the 3′-to-5′ exonuclease and polymerase activities of the End Repair Mix. A single adenosine base was then added to the 3′ ends of the repaired fragments to facilitate adapter ligation, as the adapters contained a complementary single thymine overhang. After adapter ligation, the products were purified by gel electrophoresis to remove free adapters and adapter-self-ligated products, and DNA fragments of appropriate size were selected. The adapter-ligated fragments were then enriched by PCR, with the number of cycles minimized to avoid amplification-induced errors. The resulting library was quantified using the PicoGreen method on a fluorescence spectrophotometer and quality-checked on an Agilent 2,100 to confirm fragment size distribution. Libraries were normalized to 10 nM and pooled in equal volumes, then diluted stepwise to a final concentration of 4–5 pM for sequencing. Raw sequences that passed the initial quality filter were assigned to individual libraries and samples according to their index and barcode information, and the barcode sequences were then removed. Sequence denoising or OTU clustering was performed following either the QIIME2 DADA2 pipeline or the Vsearch analysis pipeline.

For the DADA2 approach (QIIME2 version 2019.4), the following procedure was applied: first, the qiime cutadapt trim-paired command was used to trim primer fragments from the reads; reads that did not match the primer sequences were discarded. Subsequently, the qiime dada2 denoise-paired command was employed to perform quality filtering, denoising, paired-end merging, and chimera removal via DADA2. The above steps were performed separately for each library. After completing denoising for all libraries, the resulting ASV feature sequences and the ASV table were merged, and singletons ASVs (i.e., ASVs with a total abundance of 1 across all samples) were removed by default. The length distribution of the high-quality sequences was summarized using R scripts.

For the Vsearch pipeline (v2.13.4) with cutadapt (v2.3), the following steps were carried out: first, primer sequences were trimmed using cutadapt with the parameter -O 10; reads without matching primers were discarded. Paired-end reads were merged using the fastq_mergepairs module of Vsearch, and quality filtering of the merged sequences was performed with the fastq_filter module. Redundant sequences were removed using derep_fulllength, and the dereplicated sequences were clustered at 98% similarity using the cluster_size module. Chimeras were then removed using the uchime_denovo module, and a Perl script^1^ was further used to filter out chimeras from the quality-controlled sequence set, yielding high-quality sequences. Finally, these high-quality sequences were clustered at 97% similarity using the cluster_size module, and representative sequences and an OTU table were generated. Singletons OTUs (i.e., OTUs with a total abundance of 1 across all samples) and their representative sequences were subsequently removed by default.

The taxonomic composition of each sample (or group) at different phylogenetic levels was determined to obtain an overview of the community structure. For bacterial 16S rRNA genes, the Greengenes database^2^ was used as the reference by default. Taxonomic assignment was performed using the classify-sklearn algorithm in QIIME2 (Bokulich et al., 2018).^3^ Briefly, the feature sequences of each ASV or the representative sequence of each OTU were classified against a pre-trained Naive Bayes classifier using default parameters in QIIME2.

*α* diversity indices were calculated for each sample based on the distribution of OTUs across samples, and rarefaction curves were generated to evaluate whether sequencing depth was adequate. Using the non-rarefied ASV/OTU table, “the qiime diversity alpha-rarefaction” command was invoked with the parameters “--p-steps 10, --p-min depth 10, and --p-iterations 10.” The maximum rarefaction depth (--p-max-depth) was set to 95% of the sequencing depth of the sample with the lowest total read count among all samples. Ten evenly spaced depth levels were then selected between the minimum and maximum depths, and at each depth, 10 iterations of rarefaction were performed to calculate the chosen alpha diversity metrics. The mean value of the alpha diversity index at the maximum rarefaction depth was taken as the final *α* diversity estimate for each sample.

At the OTU level, distance matrices were calculated for all samples. Various unsupervised ordination and clustering methods, combined with appropriate statistical tests, were applied to assess *β* diversity differences among samples (or groups) and to evaluate the statistical significance of those differences.

At the taxonomic composition level, a range of unsupervised and supervised ordination, clustering, and modeling approaches, together with corresponding statistical tests, were further employed to assess the differences in species abundance composition among different samples (or groups).

### 2.5 Statistical analysis

All data were first preliminarily organized using Excel 2007, and then analyzed using SPSS 21.0. The experiment followed a two-factor factorial design involving feeding concentration (0.2, 0.3, 0.4%) and feeding duration (14d, 28d), giving a total of six treatment groups. An additional blank control group (without any treatment) was also included. Growth performance parameters and nutrient digestibility were analyzed using the pen as the experimental unit, whereas serum biochemical parameters were analyzed using individual piglets as the experimental unit. For the six experiment groups, a two-way analysis of variance (ANOVA) based on a general linear model (GLM) procedure of SPSS. The statistical model was:

$Y_{\mathrm{ijk}}=\mu +C_{i}+T_{j}+{\left(\mathrm{CT}\right)}_{\mathrm{ij}}+e_{\mathrm{ijk}}$

Where Y_ijk_ is the observed value, μ is the overall mean, C_i_ is the fixed effect of feeding concentration, T_j_ is the fixed effect of feeding duration, (CT)_ij_ is the interaction effect between feeding concentration and feeding duration, and e_ijk_ is the random error, assumed to be independently and normally distributed with a mean of zero and homogeneous variance N (0,σ^2^). Since the feeding concentrations (0.2, 0.3, 0.4) were equally spaced quantitative factors, polynomial contrasts were further applied in the analysis to decompose them into linear and quadratic effects, in order to evaluate the dose–response trends of each indicator with changing concentration. For post hoc multiple comparisons, Duncan’s method was employed.

Comparisons between seven groups for single factor were performed using the one-way ANOVA procedure in SPSS 21.0, followed by Duncan’s multiple range test for multiple comparisons. Statistical significance was declared at *p* < 0.05. For the effect of feeding concentration, the control group was included as the 0% concentration level in the comparison, and one-way ANOVA was performed to evaluate the effects among the four different concentrations. For the effect of feeding duration, one-way ANOVA was performed on the data from the six treatment groups to compare the two durations (14 d vs. 28 d), irrespective of concentration levels.

## 3 Results

The feeding concentration significantly affected the ADG and ADFI of weaned piglets (*p* < 0.05). The ADG and ADFI in the 0.2 and 0.3% addition groups were significantly higher than those in CON and the 0.4% addition group (*p* < 0.05) (Table 3). There were no significant differences in the F/G ratio or G/F ratio across the different concentration levels (*p* > 0.05). Feeding concentration had a highly significant main effect on FBM, ADG, and ADFI (*p* < 0.01). Polynomial trend analysis revealed that both linear and quadratic effects of feeding concentration were highly significant (*p* < 0.01) for all three parameters, indicating a curvilinear dose–response relationship between concentration and growth performance. FBM, ADG, and ADFI all exhibited a decreasing trend with increasing concentration, with the 0.2 concentration group showing the optimal growth performance among all groups. Feeding duration had a significant effect on ADFI (*p* < 0.05), but insignificant effect on F/G or G/F. The interaction between feeding concentration and feeding duration significantly affected ADG and ADFI (*p* < 0.05). During short-term feeding (14 d), ADG and ADFI were higher with low and medium concentrations of CCHA. However, after 28 d of feeding, the 0.2% concentration further enhanced both parameters, whereas the 0.3% concentration decreased ADG and ADFI in piglets. Among all experiment groups, Group IV showed the highest ADG and ADFI, which were significantly increased at a feeding concentration of 0.2% and a feeding duration of 28 days. CP digestibility in the treatment groups was significantly higher than that in the control group (*p* < 0.05), but there were no significant differences among the different concentration groups (Table 4). Analysis of linear and quadratic effects on digestibility showed that CP digestibility exhibited a rise-and-fall trend as the feeding concentration increased. Feeding duration significantly influenced the apparent digestibility of DM and CP (*p* < 0.05). Prolonged feeding duration significantly increased the digestibility of DM and CP in piglets (*p* < 0.01), and also significantly improved ASH digestibility (*p* < 0.05), but had no significant effect on EE digestibility (*p* > 0.05). The interaction between feeding concentration and feeding duration had a significant effect on the apparent digestibility of CP (*p* < 0.05), while its effects on DM, EE, and ASH digestibility were not significant (*p* > 0.05). At 14 days of feeding, the 0.4% concentration group showed the highest CP digestibility. At 28 days, CP digestibility in all concentration groups was higher than that at 14 days; among the 28-day groups, the 0.4% group had the lowest CP digestibility, while the 0.3% group had the highest. This indicates that the effect of CCHA on CP digestibility in piglets depends not only on the feeding concentration, but also requires a sufficient feeding duration to achieve the pro-digestive efficacy. However, longterm feeding of a high concentration of CCHA may inhibit CP digestion in the body. Under the conditions of Group IV and V, the DM and CP digestibility were significantly higher than those in the other five groups (*p* < 0.05).

**Table 3:** Effect of CCHA on growth performance of piglets

Items	Group	IBM(kg)	FBM(kg)	ADG(g)	ADFI (g)	F/G (g/g)	G/F(g/g)
Groups	CON	7.93±0.35	16.60±0.72^c^	309.48±32.79^d^	489.16±22.86^e^	1.59±0.10^ab^	0.63±0.04^ab^
	I	7.86±0.23	18.00±0.9^b^	362.17±31.79^bc^	584.23±17.79^bc^	1.62±0.14^ab^	0.62±0.05^ab^
	II	7.68±0.48	18.23±0.8^ab^	376.72±11.43^ab^	595.62±17.15^ab^	1.58±0.08^ab^	0.63^ab^
	III	7.82±0.32	17.44±0.83^b^	343.54±18.30^c^	556.95±7.39d	1.62±0.11^ab^	0.62±0.04^ab^
	IV	7.72±0.32	18.96±0.70^a^	401.60±27.82^a^	604.22±11.43^a^	1.51±0.12^b^	0.67±0.05^a^
	V	7.86±0.16	18.17±0.58^ab^	368.30±23.90^bc^	568.51±14.02^cd^	1.55±0.12^ab^	0.65±0.05^ab^
	VI	7.82±0.27	15.40±0.28^d^	270.73±8.78^e^	448.85±15.27^f^	1.65±0.06^a^	0.60±0.03^b^
Main effects	Level						
Fortified concentration	0	7.93±0.35	16.60±0.72^b^	309.48±32.79^b^	489.16±22.87^b^	1.59±0.10	0.63±0.04
	0.2%	7.79±0.27	18.48±0.92^a^	381.88±35.15^a^	594.23±17.67^a^	1.57±0.14	0.64±0.06
	0.3%	7.77±0.38	18.20±0.67^a^	372.51±18.40^a^	582.06±19.84^a^	1.57±0.85	0.64±0.03
	0.4%	7.82±0.28	16.42±1.22^b^	307.13±40.41^b^	502.89±57.60^b^	1.64±0.76	0.61±0.03
Feeding duration	14d	7.79±0.35	17.88±0.86	360.81±25.09	578.9±21.78^a^	1.61±0.10	0.62±0.04
	28d	7.80±0.25	17.51±1.65	346.88±60.69	540.52±69.42^b^	1.57±0.12	0.64±0.05
P value	Factor						
	Fortified concentration	0.93	<0.01	<0.01	<0.01	0.135	0.128
	Linear	0.83	<0.01	<0.01	<0.01	0.1	0.07
	Quadratic	0.76	<0.01	<0.01	<0.01	0.284	0.356
	Feeding duration	0.91	0.12	0.08	<0.01	0.30	0.268
	Fortified concentration *Feeding duration	0.47	<0.01	<0.01	<0.01	0.25	0.247

Values are means ± SD. Data for the six treatment groups (3 concentrations × 2 durations) were analyzed by two-way ANOVA (GLM) with concentration, duration, and their interaction as fixed effects; linear and quadratic polynomial contrasts were used to test dose-dependent trends. For multiple comparisons among all seven groups (six treatment groups and the CON), one-way ANOVA followed by Duncan's test was performed. In each row, means with different superscript letters differ significantly (P < 0.05); the absence of letters denotes no significant difference (P > 0.05). Significant effects of main factors and their interaction are indicated in the table.

**Table 4:** Effect of CCHA on apparent digestibility of weaned piglets (%)

Items	Group	DM	CP	EE	ASH
Groups	CON	76.56±1.03^b^	75.53±1.23^c^	77.59±1.25	33.27±0.38
	I	76.57±1.45^b^	78.67±1.66^b^	77.38±0.86	33.05±0.77
	II	76.52±1.22^b^	78.65±1.10^b^	76.97±1.47	33.13±1.06
	III	76.62±1.11^b^	79.33±1.06^b^	76.45±1.46	33.58±1.55
	IV	80.37±1.72^a^	82.50±1.26^a^	77.68±0.91	33.79±0.77
	V	81.39±1.91^a^	83.64±1.18^a^	78.03±1.62	34.09±1.73
	VI	81.64±2.19^a^	79.67±1.03^b^	77.13±1.31	34.42±0.92
Main effects	Level				
Fortified concentration	0	76.56±1.03	75.53±1.23^b^	77.59±1.25	33.27±0.38
	0.2%	78.47±2.50	80.59±2.44^a^	77.53±0.86	33.42±0.83
	0.3%	78.96±2.97	81.15±2.83^a^	77.50±1.57	33.61±1.46
	0.4%	79.13±3.10	79.50±1.01^a^	76.79±1.37	34.00±1.29
Feeding duration	14d	76.57±1.19^b^	78.89±1.27^b^	76.93±1.28	33.25±1.13
	28d	81.13±1.92^a^	81.94±2.03^a^	77.61±1.29	34.10±1.17
P value	Factor				
	Fortified concentration	0.57	0.01	0.30	0.44
	Linear	0.33	0.04	0.18	0.25
	Quadratic	0.79	0.02	0.46	0.82
	Feeding duration	<0.01	<0.01	0.13	0.03
	Fortified concentration *Feeding duration	0.59	<0.01	0.78	0.97

Values are means ± SD. Data for the six treatment groups (3 concentrations × 2 durations) were analyzed by two-way ANOVA (GLM) with concentration, duration, and their interaction as fixed effects; linear and quadratic polynomial contrasts were used to test dose-dependent trends. For multiple comparisons among all seven groups (six treatment groups and the CON), one-way ANOVA followed by Duncan's test was performed. In each row, means with different superscript letters differ significantly (P < 0.05); the absence of letters denotes no significant difference (P > 0.05). Significant effects of main factors and their interaction are indicated in the table.

The feed concentration significantly affected the levels of TP, ALB, and BUN in the piglets’ serum (*p* < 0.05) (Table 5). Dietary supplementation with CCHA increased serum TP, ALB, and BUN concentrations in piglets compared with the control group. Specifically, the highest TP and BUN levels were observed in the 0.3% concentration group, while the ALB level was highest in the group with a 0.4% addition level. The CK levels in the experimental group were significantly lower than those in CON (*p* < 0.05). Feeding duration had a significant effect on serum TP and BUN levels (*p* < 0.05) but did not significantly affect ALB and CK levels (*p* > 0.05). The interaction between feeding concentration and feeding duration significantly affected serum TP and BUN levels (*p* < 0.05), whereas no significant differences were observed in serum ALB and CK levels (*p* > 0.05). At 14 d of CCHA supplementation, the highest levels of TP and ALB were observed at the 0.4% concentration across all groups, whereas BUN peaked at the 0.3% concentration. At 28 d, within the treatment groups, the highest TP and BUN contents were found at the 0.3% concentration, while the lowest ALB content was also recorded at this concentration. When the six experimental groups were compared comprehensively, Group IV exhibited relatively higher levels of TP, ALB, and BUN.

**Table 5:** Effect of CCHA on serum biochemical indexes of piglets

Items	Group	TP/(g/L)	ALB/(g/L)	BUN/(mmol/L)	CK(U/L)
Groups	CON	53.42±2.30^d^	12.94±2.09^c^	2.47±0.44^c^	6.17±0.17^a^
	I	57.75±2.08^c^	16.48±1.63^ab^	2.83±0.43^bc^	5.46±0.30^b^
	II	62.86±1.29^b^	14.55±0.40^bc^	4.04±0.04^a^	5.28±0.16^b^
	III	65.85±1.25^ab^	17.95±0.78^a^	3.41±0.03^b^	5.33±0.29^b^
	IV	63.95±1.81^b^	16.27±0.85^ab^	3.06±0.12^bc^	5.13±0.19^b^
	V	68.29±1.29^a^	15.61±0.31^b^	3.17±0.01^b^	5.08±0.24^b^
	VI	59.69±0.94^c^	16.37±0.55^ab^	2.85±0.61^bc^	5.28±0.14^b^
Main effects	Level				
Fortified concentration	0	53.42±2.30^b^	12.94±2.09^c^	2.47±0.44^c^	6.17±0.17^a^
	0.2%	60.85±3.81^a^	16.37±1.17^ab^	2.95±0.32^bc^	5.29±0.29^b^
	0.3%	65.57±3.19^a^	15.08±0.66^b^	3.61±0.48^a^	5.18±0.21^b^
	0.4%	62.77±3.52^a^	17.16±1.05^a^	3.13±0.50^ab^	5.31±0.21^b^
Feeding duration	14d	62.15±3.80^b^	16.33±1.74	3.42±0.57^a^	5.36±0.24
	28d	63.98±3.92^a^	16.08±0.64	3.03±0.35^b^	5.16±0.19
P value	Factor				
	Fortified concentration	<0.01	0.02	0.01	0.56
	Feeding duration	0.03	0.65	0.02	0.08
	Fortified concentration *Feeding duration	<0.01	0.16	0.03	0.58

Values are means ± SD. Data for the six treatment groups (3 concentrations × 2 durations) were analyzed by two-way ANOVA (GLM) with concentration, duration, and their interaction as fixed effects; linear and quadratic polynomial contrasts were used to test dose-dependent trends. For multiple comparisons among all seven groups (six treatment groups and the CON), one-way ANOVA followed by Duncan's test was performed. In each row, means with different superscript letters differ significantly (P < 0.05); the absence of letters denotes no significant difference (P > 0.05). Significant effects of main factors and their interaction are indicated in the table.

Supplementation with CCHA led to a significant reduction in AST and ALT activities (*p* < 0.05), but had no significant effect on ALP and ACP activities (*p* > 0.05) (Table 6). With increasing feeding duration, serum ALT activity was increased (*p* < 0.01), but had no significant effect on ALP and ACP activities (*p* > 0.05). The interaction between feeding concentration and feeding duration significantly influenced serum ALT activity (*p* < 0.05). At 14 d of CCHA supplementation, ALT activity was lowest at the 0.4% concentration. However, at 28 d, ALT activity was significantly lower than that at 14 d, with the most pronounced reduction observed at the 0.2% concentration. Among all groups, Group VI exhibited the lowest ALT and AST activities, whereas insignificant differences were observed on serum ALP and ACP activities (*p* > 0.05).

**Table 6:** Effect of CCHA on serum biochemical indexes of piglets

Items	Group	AST(U/L)	ALT(U/L)	ALP(U/L)	ACP(U/L)
Groups	CON	66.10±5.76^a^	96.23±1.01^a^	23.88±1.43	20.11±0.75
	I	64.20±1.44^ab^	92.31±4.50^ab^	23.92±1.66	20.60±1.58
	II	63.23±1.93^ab^	92.94±1.91^ab^	24.08±1.05	20.32±0.80
	III	63.10±5.76^ab^	91.02±2.36b	24.32±0.60	21.27±1.74
	IV	57.97±4.16^c^	80.82±6.42^c^	24.73±1.76	20.90±1.00
	V	60.65±4.24^bc^	83.72±2.90^c^	24.91±0.30	20.67±1.18
	VI	61.01±1.79^abc^	90.68±2.60^b^	24.63±1.56	20.49±0.38
Main effects	Level				
Fortified concentration	0	66.10±5.76^a^	96.23±1.01^a^	23.88±1.43	20.11±0.74
	0.2%	61.08±4.41^b^	86.57±8.00^b^	24.32±1.59	20.75±1.19
	0.3%	61.94±3.42^b^	88.33±5.35^b^	24.50±0.83	20.49±0.92
	0.4%	62.06±4.21^b^	90.85±2.37^b^	24.48±1.07	20.88±1.20
Feeding duration	14d	63.15±4.15	93.13±3.24^a^	3.42±0.57	5.36±0.24
	28d	61.43±4.96	87.86±7.09^b^	3.03±0.35	5.16±0.19
P value	Factor				
	Fortified concentration	0.03	<0.01	0.97	0.84
	Linear	0.51	<0.01	0.84	0.85
	Quadratic	0.78	0.78	0.88	0.60
	Feeding duration	0.32	<0.01	0.31	0.934
	Fortified concentration *Feeding duration	0.35	<0.01	0.93	0.64

Values are means ± SD. Data for the six treatment groups (3 concentrations × 2 durations) were analyzed by two-way ANOVA (GLM) with concentration, duration, and their interaction as fixed effects; linear and quadratic polynomial contrasts were used to test dose-dependent trends. For multiple comparisons among all seven groups (six treatment groups and the CON), one-way ANOVA followed by Duncan's test was performed. In each row, means with different superscript letters differ significantly (P < 0.05); the absence of letters denotes no significant difference (P > 0.05). Significant effects of main factors and their interaction are indicated in the table.

The feeding concentration had no significant difference on serum immune antibody and cytokine levels (*p* > 0.05) (Table 7). Serum IgA levels were highest at the 0.2 and 0.3% addition levels. IgM and IgG levels were higher in the 0.2, 0.3, and 0.4% treatment groups compared to CON. Compared with CON, the 0.4% IL-2 treatment group showed an 11.31% increase in IL-2 levels and a 4.93% decrease in TNF-*α* levels. Serum IgG level exhibited a trend of initially decreasing and then increasing with increasing CCHA supplementation concentration (*p* < 0.05); however, the differences among concentration groups were not statistically significant (*p* > 0.05). The IL-2 concentration showed a significant linear increase in response to increasing CCHA supplementation levels. Feeding duration had a significant effect on IgM, IgG, IL-2, IL-4, and TNF-*α* (*p* < 0.05), but no significant effect on IgA (*p* > 0.05). Levels of IgG, IL-2, and IL-4 increased with longer feeding duration, while TNF-*α* was decreased. The interaction between feeding concentration and feeding duration had a significant effect on IgG production (*p* < 0.01), but there were insignificant differences in the levels of IgA, IgM, IL-2, IL-4, and TNF-α (*p* > 0.05). At 14 d, IgG level peaked at the 0.4% concentration. At 28 d, however, IgG levels were significantly elevated compared with those at 14 d, and across all six experimental groups, the highest value was observed in Group V. Compared with CON, IgA levels were elevated in experimental Groups. IgM levels in Group IV were significantly higher than those in Group I, II, and III and CON. IgG and IL-4 levels of Group V were 24.46 and 18.11% higher than CON. TNF-α levels of Group V and VI were significantly lower than CON (*p* < 0.05). Overall, Group IV showed relatively higher combined levels of serum antibodies and cytokines in piglets.

**Table 7:** Effect of Chinese herbal medicine additive on serum immunity index of Piglets

Items	Group	IgA(μg /mL)	IgM(μg/mL)	IgG(μg/mL)	IL-2(ng/L)	IL-4(ng/mL)	TNF-α(ng/L)
Group	CON	27.78±0.65	7.97±0.46d	107.82±2.82d	56.70±5.37d	42.36±4.72d	175.60±5.38a
	I	27.94±0.63	8.24±0.44cd	118.42±1.99c	58.68±4.90bd	43.06±4.81cd	173.10±2.96a
	II	28.28±0.83	8.30±0.47cd	109.34±3.47d	57.97±4.05d	42.03±4.08d	172.60±5.54ab
	III	27.83±0.81	8.38±0.47bcd	120.28±3.60c	60.33±5.83bd	44.28±5.17bcd	172.51±5.85ab
	IV	28.38±0.56	9.03±0.53a	138.64±2.68b	61.77±3.48bd	48.50±3.23abc	166.03±4.18bc
	V	28.45±0.70	8.94±0.45ab	142.73±2.80a	64.36±4.16ab	51.73±4.28a	164.25±5.37c
	VI	28.07±0.51	8.66±0.46abc	135.99±2.08b	67.54±3.00a	49.29±5.38ab	161.38±7.39c
Main effects	Level						
Fortified concentration	0	27.78±0.65	7.97±0.46	107.82±2.83b	56.70±5.37b	42.36±4.72	175.60±5.38a
	0.2%	28.16±0.61	8.64±0.62	128.53±10.79a	60.22±4.36ab	45.78±4.83	169.57±5.06ab
	0.3%	28.37±0.74	8.62±0.55	126.04±17.70a	61.16±5.15ab	46.88±6.45	168.42±6.79b
	0.4%	27.95±0.66	8.52±0.47	128.14±8.67a	63.93±5.81a	46.78±5.67	166.95±8.61b
Feeding dturation	14d	28.02±0.74	8.31±0.44b	116.11±1.35b	58.99±4.79b	43.12±4.52b	172.74±4.66a
	28d	28.30±0.59	8.87±0.48a	139.12±0.88a	64.55±4.15a	49.84±4.36a	163.88±5.79b
P value	Factor						
	Fortified concentration	0.34	0.81	0.08	0.13	0.81	0.50
	Linear	0.47	0.55	0.74	<0.05	0.59	0.24
	Quadratic	0.20	0.80	0.03	0.56	0.71	0.93
	Feeding duration	0.22	<0.01	<0.01	<0.01	<0.01	<0.01
	Fortified concentration *Feeding duration	0.88	0.41	<0.01	0.50	0.39	0.65

Values are means ± SD. Data for the six treatment groups (3 concentrations × 2 durations) were analyzed by two-way ANOVA (GLM) with concentration, duration, and their interaction as fixed effects; linear and quadratic polynomial contrasts were used to test dose-dependent trends. For multiple comparisons among all seven groups (six treatment groups and the CON), one-way ANOVA followed by Duncan's test was performed. In each row, means with different superscript letters differ significantly (P < 0.05); the absence of letters denotes no significant difference (P > 0.05). Significant effects of main factors and their interaction are indicated in the table.

The feeding concentration of CCHA significantly affected GSH-Px, T-AOC, and MDA levels (*p* < 0.05), with higher GSH-Px and T-AOC levels observed at 0.2, 0.3% concentrations, but no significant effect was detected on SOD activity (*p* > 0.05) (Table 8). MDA levels in all experimental groups were significantly lower than CON (*p* < 0.05). GSH-Px activity decreased with increasing CCHA concentration (*p* < 0.01), whereas T-AOC exhibited a trend of slowly increasing and then rapidly decreasing (*p* < 0.05), and MDA activity increased with increasing concentration (*p* < 0.01). The duration of feeding had a significant effect on GSH-Px, T-AOC, SOD, and MDA (*p* < 0.05). GSH-Px, T-AOC, and SOD levels all showed a trend toward increasing with prolonged feeding, while MDA levels decreased. The interaction between feeding concentration and feeding duration had a significant effect on T-AOC (*p* < 0.05). Under short-term CCHA supplementation, T-AOC was highest at the 0.2% concentration; however, under long-term supplementation, it peaked at the 0.3% concentration, which was significantly higher than that in all groups subjected to short-term feeding. However, it had no significant effect on GSH-Px, SOD, or MDA. Compared with CON, GSH-Px levels were elevated in all experimental groups, with Group IV showing the highest levels, an increase of 19.19% compared with CON. The T-AOC levels in Group IV, V, and VI were all higher than Group I, II, and III and CON. There were no obvious differences in SOD levels among Group IV, V, and VI (*p* > 0.05), but apparent differences were observed compared with Group I, II, III and CON (*p* < 0.05). MDA levels in all experimental groups were significantly lower than CON (*p* < 0.05), with Group IV showing the lowest levels, a 39.90% reduction compared to CON. In summary, the combination of 0.2% CCHA concentration and 28 days of feeding duration the best improvement in antioxidant indices.

**Table 8:** Effect of CCHA on serum antioxidant index of piglets

Items	Group	GSH-Px	T-AOC(Mm)	SOD/(U/ml)	MDA(nmol/L)
Groups	CON	286.71±7.04^f^	0.10±0.01^cd^	32.95±1.57^c^	8.57±0.64^a^
	I	316.07±6.72^cd^	0.11±0.01^c^	33.41±1.78b^c^	5.90±0.76^c^
	II	306.20±7.69^de^	0.09±0.03^d^	34.50±2.13^bc^	7.14±0.59^b^
	III	297.74±8.33^ef^	0.10±0.01^cd^	35.03±0.95^b^	7.50±0.59^b^
	IV	354.79±5.37^a^	0.18±0.00^b^	45.05±0.97^a^	5.27±0.36^c^
	V	336.81±6.83^b^	0.20±0.01^a^	43.40±1.51^a^	5.31±0.82^c^
	VI	323.46±9.50^c^	0.17±0.01^b^	44.25±1.03^a^	5.73±0.48^c^
Main effects	Level				
Fortified concentration	0	286.71±7.04^b^	0.10±0.01^c^	31.99±0.79^b^	8.57±0.64^a^
	0.2%	335.43±21.89^a^	0.15±0.39^a^	39.11±5.85^a^	5.58±0.64^b^
	0.3%	321.50±17.98^a^	0.15±0.60^a^	38.95±4.97^a^	6.23±1.13^b^
	0.4%	310.60±16.20^ab^	0.14±0.39^b^	39.64±4.90^a^	6.61±1.04^b^
Feeding duration	14d	306.67±10.33^b^	0.10±0.01^b^	34.49±1.32^b^	6.85±0.87^a^
	28d	338.35±15.06^a^	0.19±0.02^a^	44.23±1.57^a^	5.44±0.50^b^
P value	Factor				
	Fortified concentration	<0.01	0.03	0.42	0.02
	Linear	<0.01	0.03	0.22	<0.01
	Quadratic	0.70	0.2	0.46	0.63
	Feeding duration	<0.01	<0.01	<0.01	<0.01
	Fortified concentration *Feeding duration	0.34	<0.01	0.12	0.147

Values are means ± SD. Data for the six treatment groups (3 concentrations × 2 durations) were analyzed by two-way ANOVA (GLM) with concentration, duration, and their interaction as fixed effects; linear and quadratic polynomial contrasts were used to test dose-dependent trends. For multiple comparisons among all seven groups (six treatment groups and the CON), one-way ANOVA followed by Duncan's test was performed. In each row, means with different superscript letters differ significantly (P < 0.05); the absence of letters denotes no significant difference (P > 0.05). Significant effects of main factors and their interaction are indicated in the table.

**Figure 1:**
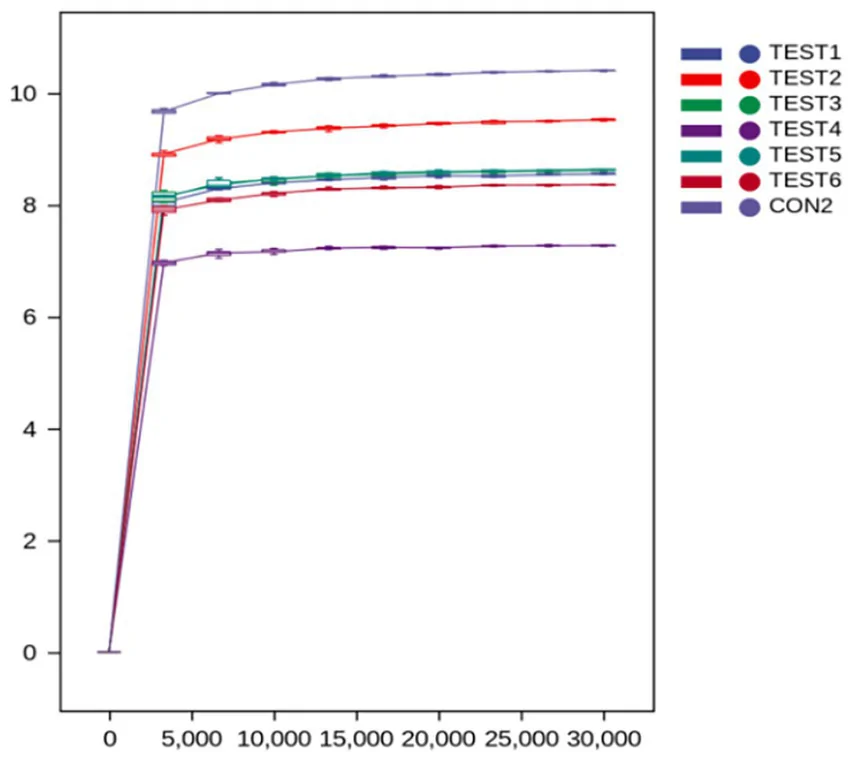
Sample dilution curve. The *x*-axis indicates the sequencing depth (number of sequences per sample), and the *y*-axis represents the median values of alpha diversity indices calculated from 10 random subsamplings at each depth. Each curve corresponds to a different group. The flattening of the curves suggests that the sequencing depth is sufficient to capture the majority of microbial diversity in the corresponding samples, as further increases in depth would yield few additional novel OTUs. In contrast, steeper curves would indicate that the alpha diversity has not yet approached saturation, implying that deeper sequencing is still needed.

In this project, community DNA fragments were sequenced using the Illumina platform with paired-end sequencing. The amplified 16S rRNA gene variable region was V3-V4. After applying DADA2 and Vsearch methods for primer removal, assembly, quality filtering, deduplication, chimera removal, and clustering, a total of 558,262 sequences were obtained. Following preliminary screening and quality control, 494,680 valid sequences were retained, representing 88.61% of the original data. The sequence lengths range from 176 to 433 bp, with an average length of 409 bp. Based on OTU classification, a total of 20,601 OTUs were identified, with an average of 2,943 OTUs per sample. The dilution curves for all groups of piglets gradually leveled off, indicating that the actual sequencing was sufficient to reflect the diversity present in the current samples, thereby demonstrating that the sequencing process complied with scientific standards (Figure 1).

**Figure 2:**
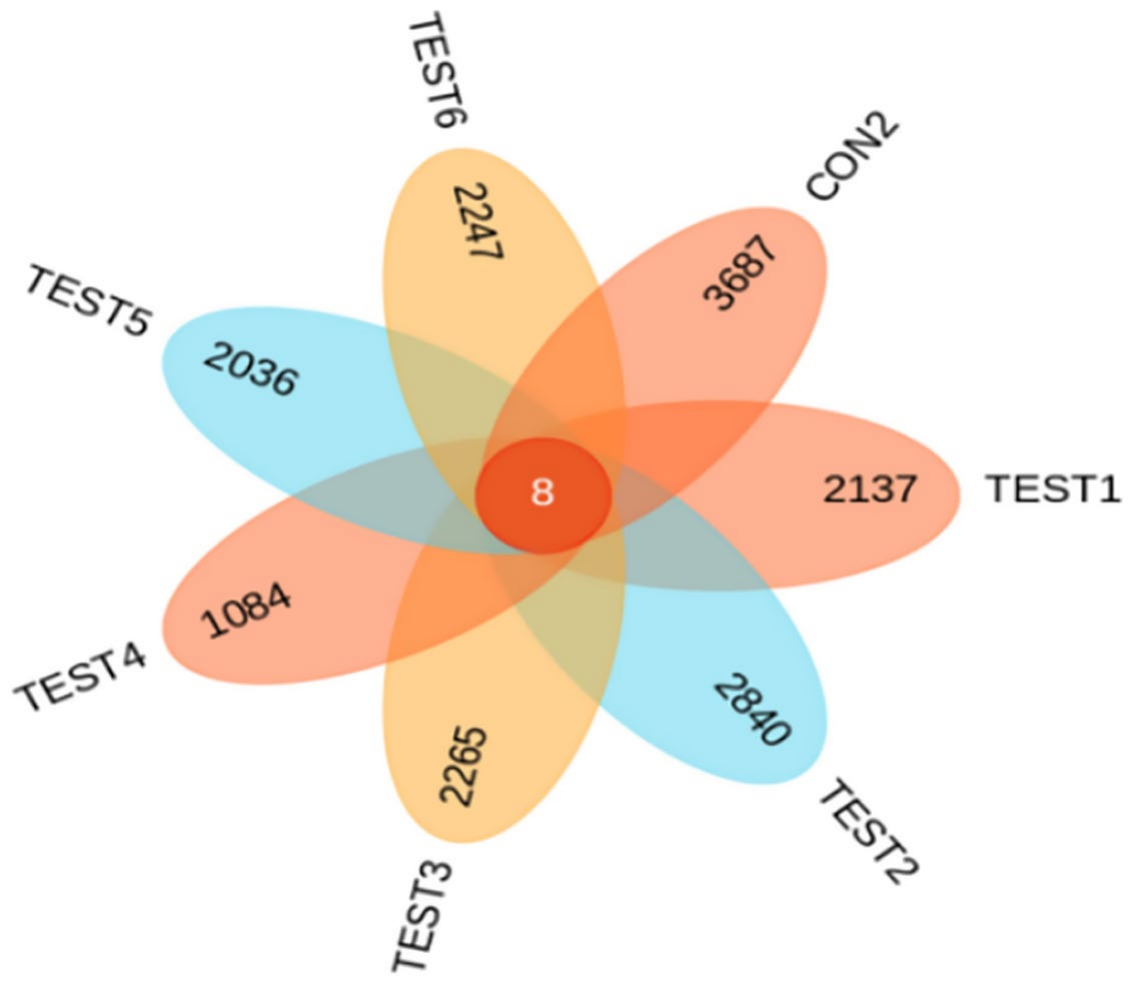
Representation of the OTUs between different groups via Venn diagram.

Analysis of the OTUs in the samples from each group revealed a total of 8 common OTUs across the 7 groups (accounting for 0.04% of the total OTUs). The number of OTUs unique to each group were as follows: CON had 3,687 (17.8% of the total OTUs), Group I had 2,137 (10.4% of the total OTUs), Group II had 2,840 (13.8% of the total OTUs), Group III had 2,265 (11.0% of the total OTUs), Group IV: 1,084 (5.3% of total OTUs), Group V: 2,036 (9.9% of total OTUs), and Group VI: 2,247 (10.9% of total OTUs) (Figure 2).

**Figure 3:**
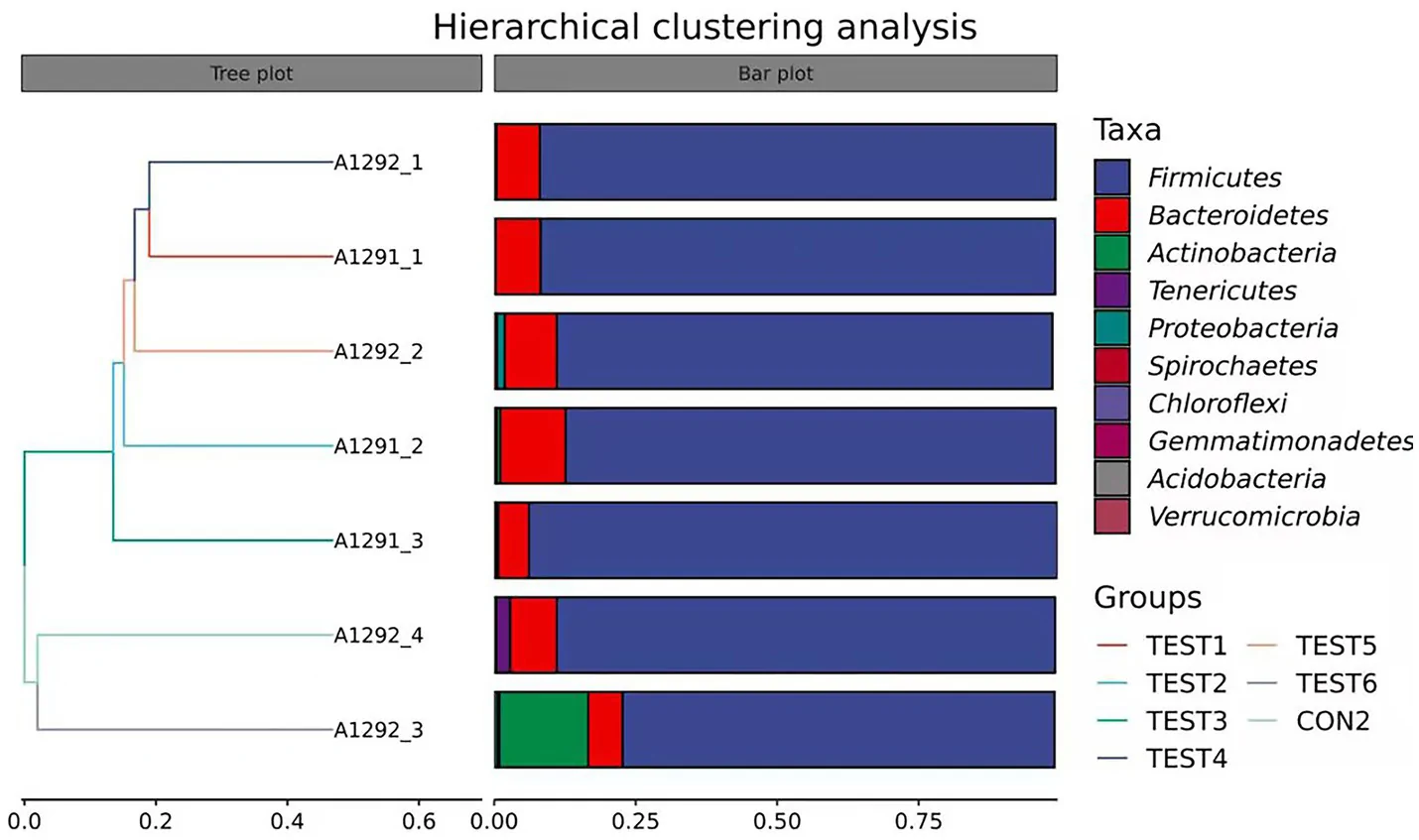
Effects of medium and short-chain fatty acid monoglycerides on phylum level of fecal microbiota in weaned piglets.

Cluster analysis at the phylum level of samples from each group (Figure 3) revealed that, the bacterial phylum distribution in CON consisted primarily of Firmicutes (88.01%), Bacteroidetes (8.19%), and Actinobacteria (0.09%). Group I consisted primarily of Firmicutes (90.88%), Bacteroidetes (7.98%), and Actinobacteria (0.05%). Group II consisted primarily of the Firmicutes phylum (86.55%), the Bacteroidetes phylum (11.56%), and the Actinobacteria phylum (0.61%). In Group III, the Firmicutes phylum accounted for 93.40%, the Bacteroidetes phylum for 5.42%, and the Actinobacteria phylum for 7.64%. In Group IV, the Firmicutes phylum accounted for 91.11%, the Bacteroidetes phylum for 7.64%, and the Actinobacteria phylum for 0.24%. And in Group V of the experiment, the Firmicutes phylum accounted for 87.53%, the Bacteroidetes phylum for 9.18%, and the Actinobacteria phylum for 1.36%. The Firmicutes phylum in Group VI accounted for 76.30%, the Bacteroidetes phylum for 6.11%, and the Actinobacteria phylum for 15.69%. Compared with CON, the abundance of Firmicutes in Group I, III, and IV increased by 2.87, 5.39, and 3.1%, respectively, while the abundance of Firmicutes in Group II, V, and VI decreased by 1.46, 0.48, and 11.71%. In Group I, III, IV, and VI, the abundance of the Bacteroidetes phylum decreased by 0.21, 2.77, 0.55, and 2.08%, respectively, while in Group II and V, the abundance of the Bacteroidetes phylum, respectively, increased by 3.37 and 0.99%. Compared with CON, the abundance of Actinobacteria increased by 0.52, 0.32, 0.15, 1.27, and 15.60% in Group II, III, IV, V, and VI, while it decreased by 0.04% in Group I.

**Figure 4:**
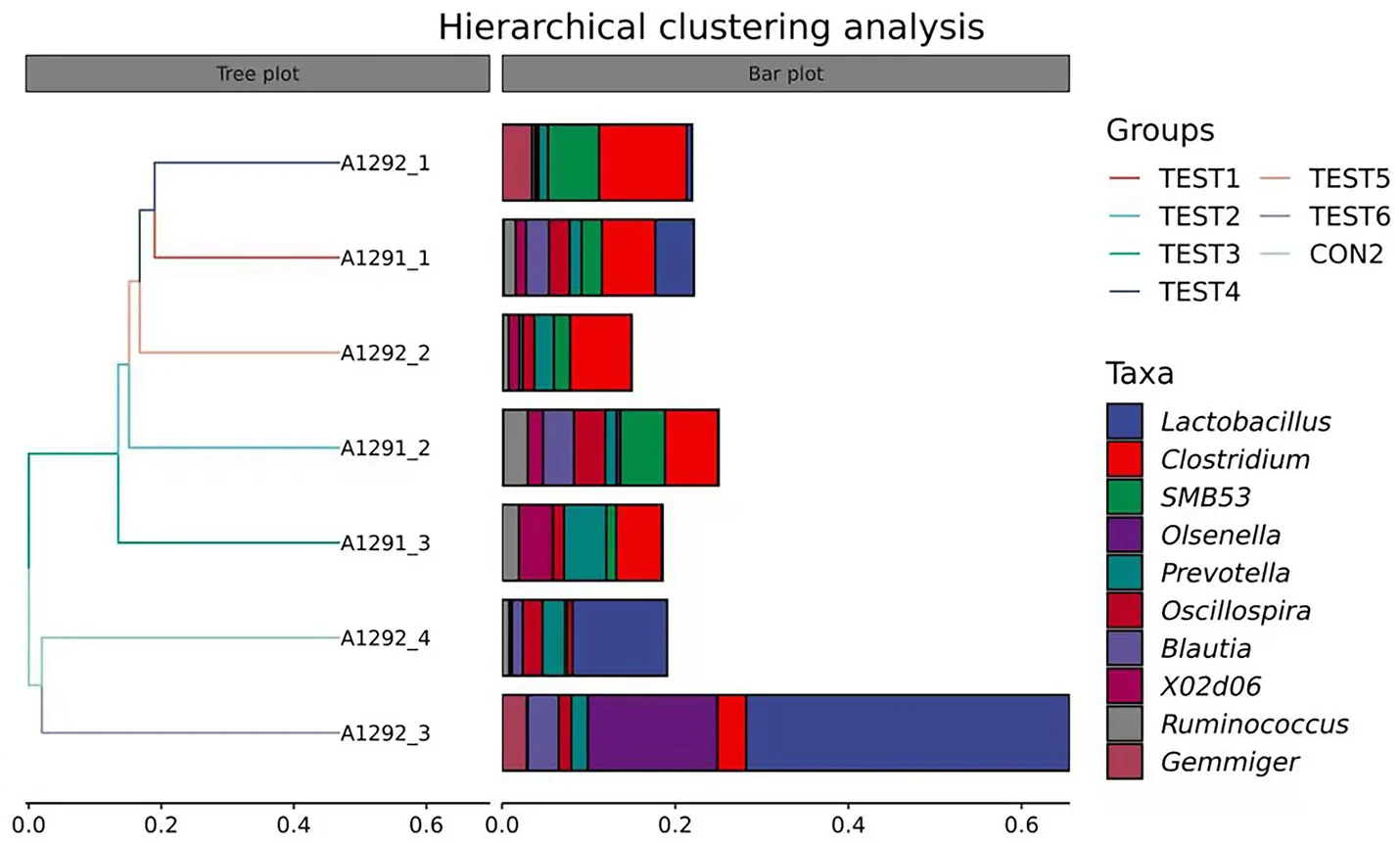
Effects of medium and short-chain fatty acid monoglycerides on the fecal microbiota at the genus level in weaned piglets.

Cluster analysis at the genus level of samples from each group (Figure 4) revealed that, the bacterial genus distribution in CON was primarily composed of Lactobacillus (10.93%), Prevotella (2.60%), and Oscillospira (2.29%). The bacterial genus distribution in Group I was primarily Lactobacillus (4.48%), Clostridium (6.16%), SMB53 (2.33%), Oscillospira (2.38%), and Blautia (2.65%). Group II consisted of Clostridium (6.09%), SMB53 (5.16%), Oscillospira (3.62%), and Blautia (3.57%). Experimental Group III consisted of Clostridium (5.25%) and Prevotella (4.88%). Experimental Group IV contained Clostridium (10.11%), SMB53 (5.90%), and Gemmiger (3.45%). Group V: Clostridiaceae_Clostridium (7.09%), Prevotella (2.27%), SMB53 (1.85%). In Group VI, the bacterial genera included Lactobacillus (37.99%), Clostridium (3.43%), Olsenella (14.94%), and Blautia (3.56%).

**Figure 5:**
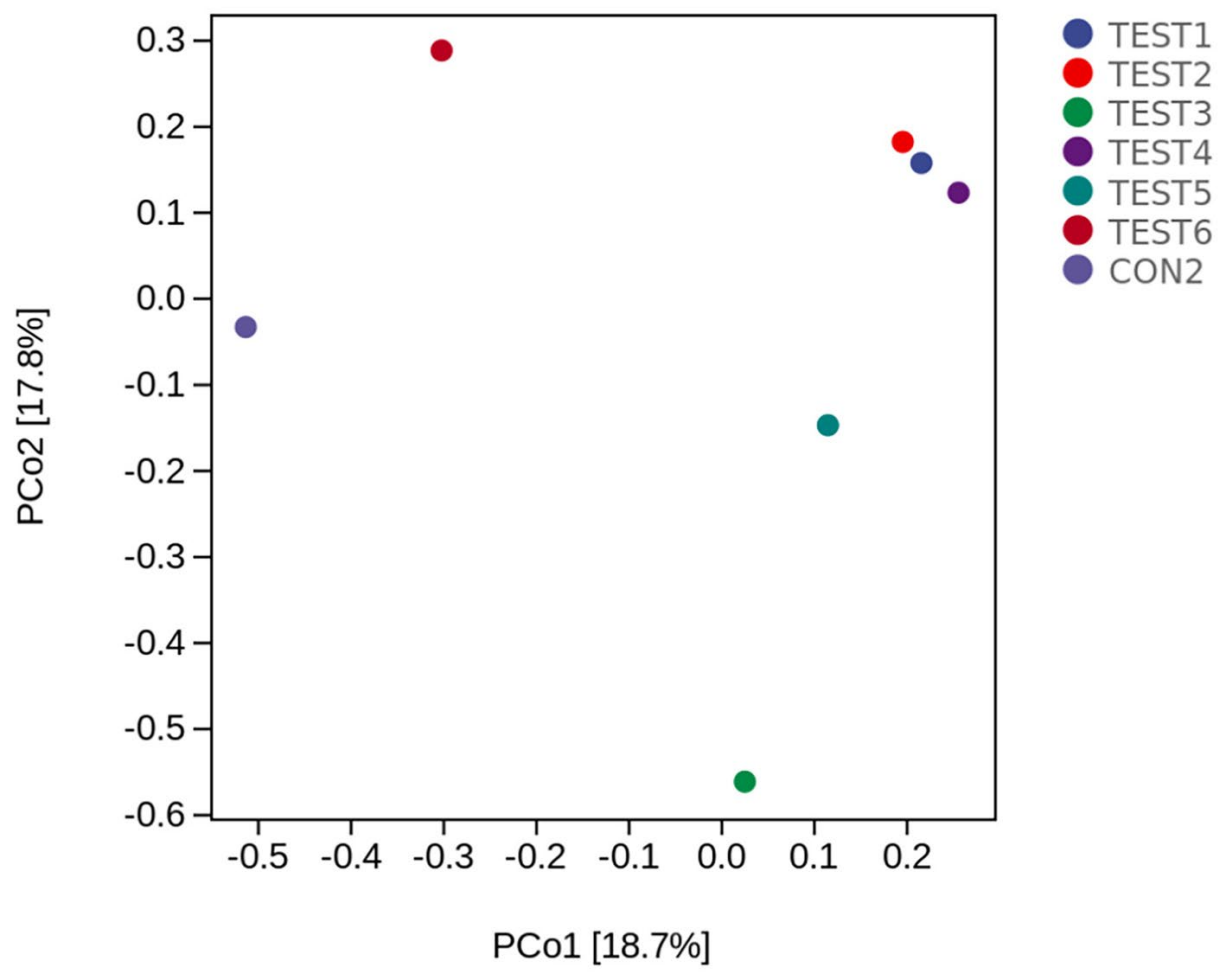
PCoA diagram of fecal microbial OTU levels in different treatment groups.

**Figure 6:**
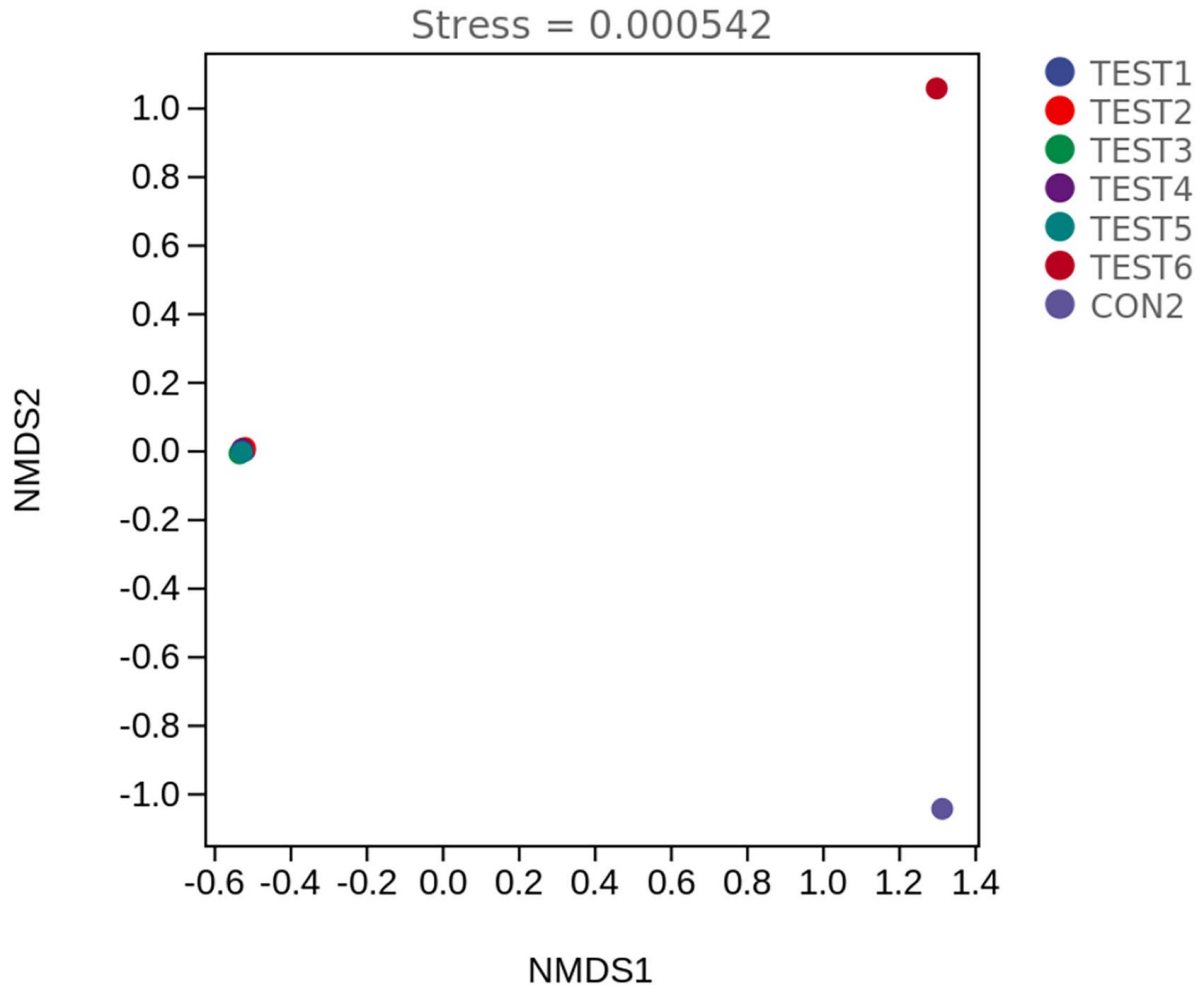
NMDS analysis of microbial community structure based on Bray-Curtis distances.

**Figure 7:**
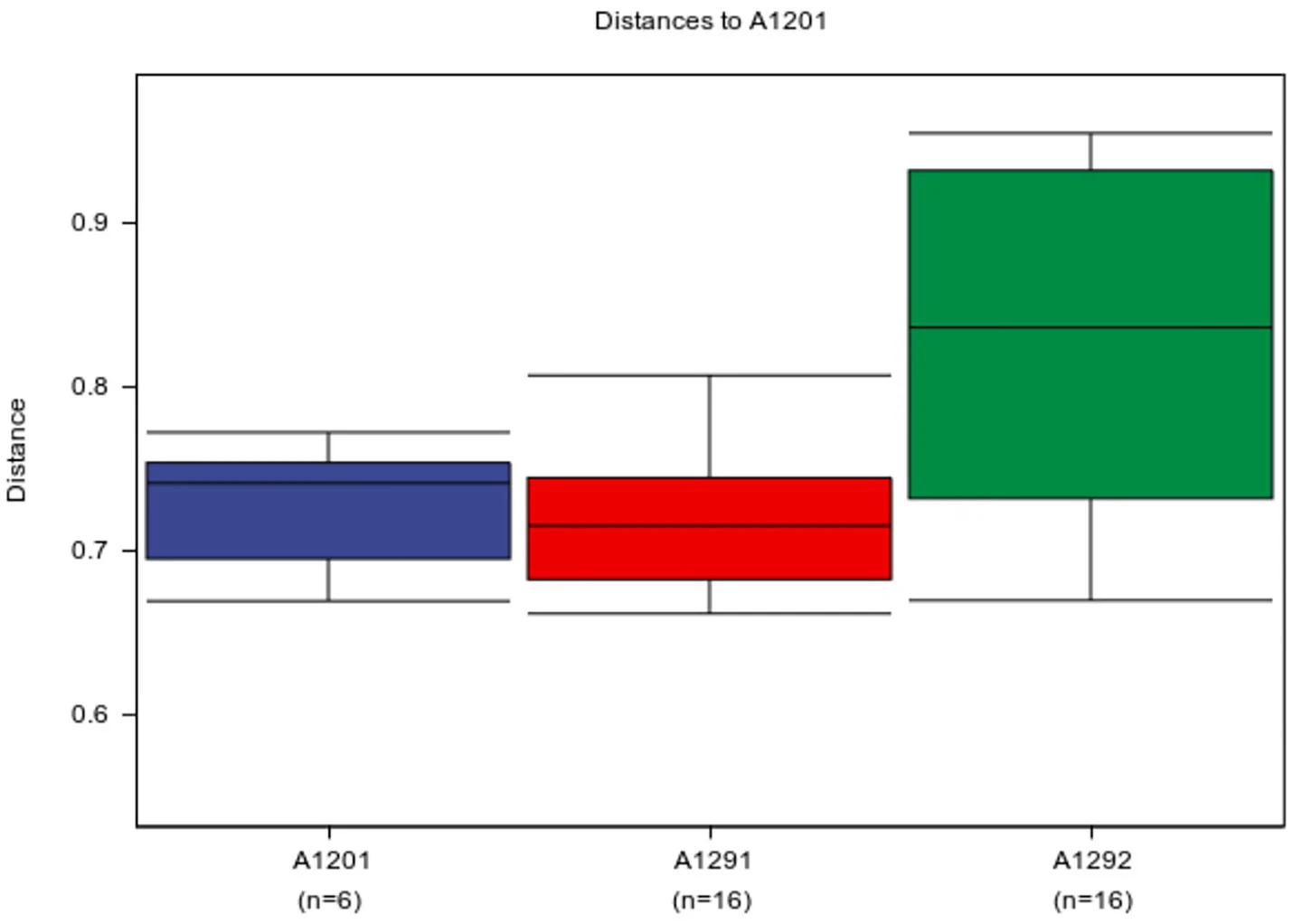
PERMANOVA results for intergroup differences in microbial community structure.

Principal Coordinate Analysis (PCoA) is a classic form of unconstrained multidimensional scaling (cMDScale). It projects the sample distance matrix onto a lower-dimensional space while maximizing the preservation of the original distance relationships among the samples. Each point in the figure represents a sample, and points of different colors indicate different groups. The percentages in parentheses on the axes represent the proportion of sample variation (distance matrix) that the corresponding axis explains. The closer projected distances of two points are on the axes, the more similar community compositions of these two samples are in the corresponding dimension. There were significant differences between some groups, while the intergroup distances were smaller for samples in Group I, II, and IV (Figure 5). The microbial community composition within these groups was highly similar, with minimal variation. In contrast, the intergroup distances were larger between CON and the other three experimental groups, indicating significant differences in microbial community composition. NMDS analysis based on Bray-Curtis distances revealed no clear separation between the experimental groups and the control group, as all samples intermingled on the ordination plot (Figure 6). The low stress value (0.000542) confirmed that the two-dimensional configuration reliably captured the underlying dissimilarities among samples. The PERMANOVA results revealed no statistically significant differences in intergroup microbial community structure (*p* > 0.05) (Figure 7), suggesting that *β*-diversity remained largely unchanged and that no systemic alteration in the overall microbial community composition occurred.

## 4 Discussion

The application and research of herbal additives in livestock production are on the rise, owing to their ability to improve the growth performance of farm animals, enhance their immune and antioxidant capacities, and reduce stress. Weaned piglets are under severe stress after weaning and are prone to issues such as growth retardation and weakened immunity. At this stage, adding herbal preparations to their diet is particularly important, as it helps alleviate stress, improve production efficiency, and minimize economic losses. Research by Deng (16) indicates that adding 0.5% or 1% of a compound feed additive containing *Codonopsis pilosula*, *Atractylodes*, and *Alisma orientale* to the diet of weaned piglets significantly increased their ADG gain and ADFI. Adding 0.2% or 0.3% of compound Chinese herbal formulations, such as *Astragalus*, *Codonopsis*, *Forsythia*, and *Atractylodes*, to the diet can significantly increase the ADG and ADFI of weaned piglets (17). The study by Lin (18) showed that adding a compound Chinese herbal preparation (containing *Coptis*, *Scutellaria*, *Phellodendron*, *Astragalus*, *Atractylodes*, etc.) to the diet of weaned piglets significantly improved their growth performance. Plant extracts can effectively enhance the growth performance of broiler chickens and reduce the feed conversion ratio (FCR) (19). Under the conditions of this experiment, the best results for ADG and ADFI were observed in Group IV, where the CCHA was added at a concentration of 0.2% and fed for 28 days. The ADG and ADFI were significantly affected by the interaction between feeding concentration and feeding duration, indicates that the optimal growth performance (Group IV: 0.2% + 28 d) reflects a synergy where the bioactive compounds of CCHA require sufficient time to modulate gut microbiota and digestive function, and neither optimal concentration alone nor prolonged duration alone could achieve comparable effects. This findings is consistent with the results of previous studies. Since this experiment did not directly investigate the mechanism of action of CCHA, the above results might partially explain that the function of *Atractylodes macrocephala*, *Codonopsis pilosula*, and *Forsythia suspensa*, which invigorating the spleen and strengthen the stomach, tonifying qi and lifting yang, and drying dampness and promote diuresis, have enhanced the appetite of weaned piglets, improved the efficiency of nutrient utilization, and thereby promoted their growth and development (20, 21).

Chinese herbal medicines contain a variety of nutrients and bioactive compounds essential for animal growth and development. These components can stimulate the secretion of digestive glands and enhance intestinal peristalsis, thereby promoting the body to produce more digestive enzymes and improving the digestibility of nutrients (22, 23). Plant extracts can significantly improve the apparent digestibility of DM, CP, and EF in goats (24). *Lonicera flos* and turmeric extracts can enhance the activity of digestive enzymes in the gastrointestinal tract and improve the digestibility of nutrients (25). Coffee grounds extract and the traditional Chinese medicine formula Sini Tang can effectively increase intestinal villus height and crypt depth, thereby promoting the digestion of nutrients in broiler chickens (26, 27). Research indicates that feeding a composite extract of hawthorn and Chinese yam can improve the apparent digestibility of nutrients such as CP, ASH, and EE in weaned piglets (28). *Codonopsis pilosula polysaccharides* can promote the digestion and absorption of nutrients by stimulating the secretion of appetite-related hormones (29). In this experiment, the apparent digestibility of ASH and CP was significantly higher than CON and showed an increasing trend with prolonged feeding duration. These results are consistent with findings of previous studies. The significant feeding concentration × feeding duration interaction for CP digestibility suggests that the efficacy of CCHA in enhancing protein digestion is time-dependent, this might be related to the adaptability of intestinal proteases or microbiota to the plant-based active components. The improvement in the apparent digestibility of nutrients is likely related to the functions of *Atractylodes macrocephala* and *Codonopsis pilosula* in CCHA to tonify the spleen, strengthen the stomach, and promote water metabolism (30, 31). Furthermore, the mechanism by which plant active components affect nutrient digestibility still requires further investigation.

Biochemical indicators in the blood serve as crucial reference standards for evaluating the health status and metabolic processes of animal bodies, and reflecting the functional states of internal organs. Serum TP and ALB levels are associated with protein synthesis and metabolism in piglets. High TP and ALB concentrations indicate high efficiency in protein absorption and utilization (32). The addition of herbal dietary supplements to broiler chicken diets increases serum TP and ALB levels (33). Under the conditions of this experiment, Group V had the highest TP content and Group III showed the highest ALB content. The BUN levels in all experimental groups were higher than CON, indicating that the CCHA enhanced protein catabolism, leading to the increase of TP levels in the body and a corresponding rise in BUN levels. These findings are consistent with the results reported by Li (34). Ephedra extract has a significant effect on reducing ALT and AST levels, and the addition of a compound of *Isatis* root and *Astragalus* to the diet also reduces serum transaminase activity (35, 36). In this study, AST and ALT levels in the experimental groups were lower than those in CON. A significant interaction between CCHA feeding concentration and feeding duration was observed for serum ALT activity. This indicates that the reduction in ALT was not merely a dose-dependent effect but was progressively amplified with prolonged feeding, suggesting that the liver-protective effects of the bioactive components from *Coptis, Lonicera, Ephedra, and Forsythia* in CCHA requires sufficient time for the cumulative absorption and metabolic conversion. Which of the bioactive substances in CCHA can affect the activities of ALT and AST, and how they exert their effects, still require further research to prove.

IgA, IgM, and IgG are important immunoglobulins found in mammalian serum. Their primary biological functions include antigen binding, complement activation, and participation in the body’s immune processes. Maintaining high levels of immunoglobulins in piglets during the early weaning period is crucial for the subsequent growth and development of piglets. Studies have shown that adding *Astragalus* to the diet of weaned piglets increases the levels of immunoglobulins in their serum and intestinal mucosa. Furthermore, the gut microbiota influenced by calycosin (a flavonoid from *Astragalus*) is strongly correlated with the levels of cytokines and immunoglobulins in the piglets’ serum (37). The compound Chinese herbal formula containing *Codonopsis*, *Poria*, and *Atractylodes* can increase serum levels of IgA, IgG, and IgM in Hu sheep (38). A dietary supplement composed of *Coptis*, *Pulsatilla*, and *Gentiana* can elevate the immunoglobulin levels in goose serum and enhance their immunity (39). In this study, serum IgM and IgG levels in all experimental groups were significantly higher than those in CON, which is consistent with the previous research findings. When foreign antigens enter an animal’s body and trigger inflammation, the body’s immune and non-immune cells secrete cytokines such as TNF-*α*, IL-2, and IL-4, which play a role in the differentiation and development of immune cells. Research has found that *Rhizoma Atractylodis macrocephalae* extracts exhibit excellent antioxidant and anti-inflammatory effects. It is commonly used to treat various chronic diseases as they can reduce serum levels of interleukins and TNF-α (30). A mixture of forsythia and lsatis root can reduce the levels of IL-6 and TNF-α in broiler serum and alleviate high fever (40). In this experiment, the levels of IL-2 and IL-4 in the experimental groups were both higher than CON, while TNF-α levels were lower than those in CON. However, the levels of IgA, IgM, IL-2, IL-4 and TNF-α are only affected by the cumulative effect of feeding duration, while IgG is influenced by the interaction effect of feeding concentration and feeding duration. Which indicates that the effect of concentration on IgG production was not uniform across different feeding durations. Specifically, the enhancing effect of CCHA on IgG became more pronounced with extended feeding duration, suggesting that an adequate duration (28 d) was required for the concentration-dependent immunostimulatory effects to be fully manifested. This interaction likely reflects the time-dependent accumulation of bioactive compounds. In contrast, IgM and IgA levels, as well as the cytokines measured in this study, did not exhibit such interaction, implying that these parameters may be more directly responsive to the mere presence of CCHA rather than to the cumulative effect of prolonged administration. The significant interaction for IgG explains the importance of both optimal concentration selection and sufficient feeding duration when designing CCHA supplementation strategies in practical production. These findings indicate that some active ingredients in CCHA may be the crucial factors enhance the immune capacity of weaned piglets. Since we did not re-measure the active ingredients of CCHA during the experiment, the above information is merely a speculation. Further research is needed in the future.

In the metabolic processes of animals, the production and elimination of free radicals exist in a dynamic equilibrium. When animals are subjected to stress, excessive free radicals are produced, which can damage tissues and cells in the body. SOD, CAT, and GSH-Px are key enzyme systems responsible for eliminating free radicals, thereby reducing the damage caused by free radicals to body cells (41). Herbal additives have significant antioxidant effects on animal bodies, adding Guizhi Lizhong Decoction to the diet can effectively increase SOD levels in piglets and reduce oxidative stress (42). The addition of a Chinese herbal supplement composed of *Astragalus, Codonopsis*, and *Magnolia* officinalis to the diet of weaned piglets can be markedly increased SOD and GSH-Px activity and reduced MDA levels (3). The Flavonoid extracts causes a decrese of MDA levels in broiler chicken serum while increasing the contents of T-AOC, SOD, and CAT, effectively enhancing the body’s antioxidant capacity (43). Among the active components of ephedra, the levels of total phenolics and total flavonoids are highly correlated with its antioxidant activity, consequently, ephedra exhibits strong antioxidant properties (44). In this study, the concentration and feeding duration of CCHA exerted differential effects in antioxidant parameters. The activities of GSH-Px and T-AOC in the experimental groups were significantly affected by both feeding concentration and feeding duration, whereas SOD activity was signficantly influenced only by feeding duration. Additionally MDA levels were apparently reduced by increasing concentration and prolonged feeding duration, but no significant interaction was observed between the two factors for MDA. Notably, T-AOC was the sole parameter showing a significant feeding concentration × feeding duration, which indicates that the synergistic effect of the two factors was specifically manifested in T-AOC. The differential responsiveness of these parameters may reflect their distinct regulatory mechanisms. The time-dependent increase in SOD activity, independent of concentration, suggests that the upregulation of SOD may be mediated through a cumulative effect of continuous CCHA intake, rather than a direct dose-dependent action of specific phytochemicals. In contrast, the concentration-dependent improvements in GSH-Px and T-AOC suggest that these parameters are more directly responsive to the abundance of antioxidant-active compounds (e.g., flavonoids, polysaccharides, and saponins) present in CCHA. The significant interaction effect on T-AOC further implies that the activity enhancement requires both an optimal concentration (0.2%) and a sufficient feeding period (28 d) to allow for the accumulation and synergistic action of multiple active components. However, it should be acknowledged that the present study did not measure the specific phytochemical composition of CCHA, therefore, the proposed mechanistic interpretations remain speculative and warrant further investigation.

One limitation of this study is the absence of baseline blood samples before the experiment. Without baseline measurements, we are unable to assess within-animal changes over time. However, the randomized allocation of piglets to treatment groups minimized the risk of systematic baseline differences among groups. Future studies should consider collecting blood samples at both the beginning and the end of the trial to enable a more comprehensive evaluation of the treatment effects.

The animal intestine is not only an organ responsible for digestion and absorption, but also the body’s largest defense organ. The barrier function of the intestinal mucosa constitutes a crucial component of the body’s mucosal immunity, and gut microbiota can indirectly influence the immune status of the organism by affecting the health of the intestinal mucosa (45). Herbal additives and fermented herbal medicines can correct imbalances in the gut microbiota and increase the number of beneficial bacteria in the intestines of weaned piglets (46). The fermented herbal preparation containing astragalus, licorice, and purslane affects the microbial *α*-diversity index in the colons of weaned piglets and promotes gut health (47). Atractylodes polysaccharides used to treat colitis in mice, can alleviate dysbiosis of the gut microbiota and maintain the integrity of the intestinal barrier (48). Numerous studies have reported that the dominant bacterial phyla in piglet feces are the Firmicutes and Bacteroidetes, which is consistent with the results of this study. In Group I, III, and IV, the abundance of Firmicutes increased by 2.87, 5.39, and 3.1%, respectively, compared to CON. However, the increased abundance of Firmicutes exhibited group-dependent changes and should not be directly interpreted as an overall increase in beneficial intestinal bacteria. At the genus level, Lactobacillus was the dominant genus in both groups I and VI, which is consistent with findings from a previous study on the combined supplementation of *Astragalus* polysaccharides and ginseng polysaccharides in weaned piglets (49). In group VI, the decreased abundance of Firmicutes was accompanied by a marked enrichment of Lactobacillus. Nevertheless, since the PERMANOVA test (*p* > 0.05) for *β*-diversity did not reveal any statistically significant differences, these alterations might be confined to specific taxonomic levels (phylum/genus) and have not yet reached the threshold required to drive a systemic shift in the overall community structure. This result suggests that CCHA exerts a ‘selective regulatory’ effect on the gut microbiota, preferentially influencing the colonization and proliferation of particular functional genera (e.g., Lactobacillus), while having only a minor impact on the core architecture of the entire microbial community.

The other limitation of this study is that we did not conduct a formal cost–benefit analysis or evaluate the economic feasibility of CCHA supplementation at different inclusion levels. While our results demonstrate that 0.2% CCHA combined with a 28-day feeding period yielded the most favorable overall outcomes, the absence of detailed economic data limits our ability to provide precise economic guidance for producers. Future studies should incorporate comprehensive economic evaluations, including cost–benefit analysis, to translate these biological effects into practical recommendations for the swine industry. Additionally, trials with a wider range of CCHA concentrations (e.g., 0.1 to 0.5%) would help further refine the optimal inclusion rate and better define the cost-effectiveness threshold.

## 5 Conclusion

The CCHA studied in this experiment significantly improved the growth performance, apparent digestibility, serum biochemical parameters, immune capacity, and antioxidant indices of weaned piglets. The CCHA tested in this experiment significantly improved the growth performance, apparent nutrient digestibility, Specifically, the group supplemented with 0.2% CCHA for 28 days exhibited the highest ADG and ADFI, the greatest digestibility of DM and CP, relatively higher serum TP content, the lowest ALT and AST activities, elevated levels of serum antibodies and immune factors, and the best antioxidant indices, as reflected by T-AOC, GSH-Px, and SOD activities. Additionally, CCHA modulated the abundance of dominant bacteria, including Firmicutes and Lactobacillus, thereby exerting a certain regulatory effect on the intestinal microecology of piglets.

## Data Availability

The data analyzed in this study are available in the OMIX database (https://ngdc.cncb.ac.cn/omix) under accession OMIX019826.

## Footnotes

1 Available at: https://github.com/torognes/vsearch/wiki/VSEARCH-pipeline.

2 Release 13.8, https://ftp.microbio.me/greengenes_release/gg_13_8_otus/taxonomy/.

3
https://github.com/QIIME2/q2-feature-classifier

## Glossary

CCHA: Compound Chinese Herbal Additives
ADG: Average Daily Gain
ADFI: Average Daily Feed Intake
IBM: Initial Body Mass
FBM: Final Body Mass
F/G: Feed-to-gain Ratio
G/F: Gain-to-feed Ratio
DM: Dry Matter
CP: Crude Protein
EE: Ether Extract
ASH: Crude Ash
TP: Total Protein
ALB: Albumin
BUN: Blood Urea Nitrogen
UREA: Serum Urea
CK: Creatine Kinase
AST: Aspartate Transaminase
ALT: Alanine Transaminase
ALP: Alkaline Phosphatase
ACP: Acid Phosphatase
IgA: Immunoglobulin A
IgM: Immunoglobulin M
IgG: Immunoglobulin G
IL-2: Interleukin-2
IL-4: Interleukin-4
IFN-γ: Interferon-γ
TNF-α: Tumor Necrosis Factor-α
GSH-Px: Glutathione Peroxidase
T-AOC: Total Antioxidant Capacity
SOD: Superoxide Dismutase
MDA: Malondialdehyde
